# Continued Fever.—The Type of Disease Now Prevalent—Cases.—Injurious Effects of Evacuants in the Early Stage, &c.

**Published:** 1865-10

**Authors:** N. S. Davis

**Affiliations:** Prof. of Clinical Medicine, &c., Chicago, Ill.


ARTICLE XXXV.

### CONTINUED FEVER.—THE TYPE OF DISEASE NOW PREVALENT.—CASES.—INJURIOUS EFFECTS OF EVACUANTS IN THE EARLY STAGE, &c.; BEING THE ABSTRACT OF A CLINIC IN THE MEDICAL WARDS OF THE MERCY HOSPITAL.

BY N. S. DAVIS, M.D., Prof. Clinical Medicine, &c., Chicago, Ill.

GENTLEMEN:—The case before you is a female, aged about 20 years, a native of Ireland, and a resident of this country only a few weeks. Some ten days since, she came to my office, complaining of some pain in her head, with some pain also in the back and limbs; a sense of oppression or tightness across the chest; looseness of the bowels; loss of appetite, and lightness or dizziness in the head. She presented an anxious expression of countenance; dryness of the lips; a thick, dirty white coat on the middle and posterior part of the tongue; some tremulousness of both tongue and lips; skin dry, harsh and congested; pulse soft, weak, and about 110. She had been complaining about one week, during which time she had taken two doses of "salts and senna," each operating freely as physic, and, during the two days that had intervened since the second dose, had continued to have four or five copious, thin, intestinal discharges each day. I advised the patient to go home, keep quiet, take bland nourishment, and sufficient anodynes to control the intestinal evacuations, and informed her that she had an attack of continued fever. About one week after she was brought to the hospital. She is, consequently, now near the end of the second week of her fever.

The symptoms I wish you to notice carefully, at present, are the following:—The whole cutaneous surface is dry, harsh, and warmer than natural; the face slightly redder than natural; eyes suffused, and expression dull; position dorsal; respiration short, accelerated, and accompanied by strongly marked dry bronchial râles all over the chest, and occasional rough cough; dulness on percussion over the lower and posterior part of the chest, but very little expectoration; pulse 115 per minute, small and weak; abdomen tumid, inelastic, but only moderately tympanitic; three or four thin, brown, intestinal evacuations each day; tongue and lips dry, dark red, and tremulous; slight subsultus; and hearing dull.

Such are the more important symptoms presented by the patient; and what are the pathological conditions indicated by them? For symptoms are valuable to a rational practitioner, only as they indicate, more or less clearly, ths nature, location, stage of progress, and tendencies of such pathological or morbid conditions as actually exist. In the patient before us, the somnolency, the dulness of hearing, the sub-sultus, and the frequent mental wandering point directly to a strongly depressed condition of the nervous functions. The dryness of the skin, the lips, the mouth, the mucous surface of the bronchial tubes, and the scantiness of urine, equally indicate a depressed or retarded condition of all the secretory functions. The moderately congested and livid hue of the skin, the congested condition of the lower and posterior portions of the lungs, as indicated by dulness on percussion, and the soft, weak pulse indicate general feebleness of capillary circulation.

Thus, the three great functions of innervation, circulation, and secretion are much impaired, producing all the phenomena of a low type of fever, while in this patient the enfeebled capillary circulation in the lungs has led to a serious degree of infiltration of the pulmonary tissue, and in the mucous membrane of the lower half of the intestinal tube to a scarcely less dangerous degree of effusion constituting the liquid stools and a tendency to softening or ulceration. Such are the proximate pathological conditions presented by the patient.

But a further inquiry arises, of great interest, but less easily answered, namely:—What causes the well-marked depression of all the functions just named? Is it the action of some morbific agent or fever poison, primarily on the nervous centres depressing nerve force, and through this influencing the vascular and secretory functions? This was essentially the doctrine of Cullen, and of a majority of writers on practical medicine, from his day to the present time. A more modern, and somewhat popular doctrine at the present time, alleges that the supposed fever poison acts primarily upon the constituents of the blood by a zymotic or septic influence, and that all the important functional disturbances are consequent on the deteriorated condition of that fluid. A few, like Virchow and Addison, who make all the phenomena of life depend on cell action, would make the phenomena of fever depend on the primary modifying influence of the fever poison on the functions of the cells, both in the blood and the tissues. Careful study and extensive clinical observation will show you that all these theories are defective and unsatisfactory.

My own opinion has often been expressed, both here and in the lecture room. It is, that the initial steps of morbid action, constituting fever, are neither in the nervous, secretory, or vascular structures or functions separately, but in the elementary properties common to all the tissues. But it was no part of my purpose, at this clinic hour, to dilate upon the essential pathology of fevers. The symptoms of the patient lying before us are of the most serious import. The universal depression of vital properties, with the extensively engorged condition of the lungs, rendering respiration imperfect, while the frequent intestinal evacuations still continue, renders the prognosis extremely unfavorable. The deteriorated condition of the blood always existing in typhus, is here increased by the diminished interchange of carbonic acid gas for oxygen through the lungs, and there is danger of a suspension of life before any change can be effected by which a more healthful impression of the blood upon the capillary and secretory structures can be made.

Still, the rational indications for treatment are plain. They are to repress the intestinal evacuations, to arrest the further engorgement of the pulmonary tissues, and to sustain the functions of the nervous and vascular systems. To accomplish the first, there is probably no remedy more efficacious than oil of turpentine combined with a certain proportion of tincture of opium. It not only exerts a peculiar action on the mucous surfaces of the intestines, by which the tone or contractility of the capillaries is increased and the accumulation of blood consequently diminished, but it also increases the activity of the whole capillary vascular system. Hence, it not only fulfils the local indication, but aids materially in accomplishing the third object named. To devise remedies that will relieve the extreme congestion in the lungs and promote the reäbsorption of the dark blood infiltrated into the posterior and lower parts of these organs is no easy task. The immediate cause of the infiltration or engorgement is a failure in the susceptibility of the capillaries and their consequent dilatation. When this state of bronchial and pulmonary congestion commences early in the progress of the fever, I have found no remedy for its relief so reliable as small doses of tartrate of antimony and potassa, given in combination with opium sufficient to prevent any danger of acting on the bowels. Given in the early stage, it lessens the general febrile action, promotes perspiration, and renders respiration and expectoration much more free. But when the patient, as in this case, has already advanced to that state when the pulse is small and weak, the skin relaxed, the abdomen tympanitic and bowels very loose, with much dulness on percussion over the middle and lower part of the lungs, and somnolency, you can expect no benefit from so direct a sedative as antimony. The principal reliance must then be placed on such remedies as tend to sustain the sensibility and action of the nervous and vascular systems, and prevent the further deterioration of the blood. We will give this patient ten drops each of oil of turpentine and tincture of opium, in the form of an emulsion, every four hours, and the sixteenth of a grain of strychnine with four drops of nitric acid dissolved in sweetened water, between. We will also dissolve two drachms each of chlorate of potassa and gum Arabic in a tumblerful of cold water, and let the patient take a tablespoonful every three hours. The patient must also be fed regularly, every two hours, with two or three tablespoonfuls of a porridge made of sweet milk and wheat flour or oatmeal. Seeing the sub-sultus, the weak pulse, the oppressed breathing, the dingy or leaden hue of the skin, and the mental dulness exhibited by the patient, you ask why I do not order brandy or other alcoholic stimulant? I answer, that nearly thirty years of careful observation at the bedside of the sick has satisfied me that under such circumstances strychnine is a far more reliable remedy for sustaining the nervous functions than alcohol, while the effects of the latter, in diminishing the decarbonization of the blood, makes it positively detrimental to the already seriously embarrassed condition of the lungs.

Before leaving the subject suggested by this patient, I must add a few words in relation to the character of the cases of continued fever prevalent in this city the present season, and a caution in regard to the use of harsh evacuants in the early stage. Since the epidemic of erysipelas, in the fall and winter of 1863, a large amount of our attacks of continued fever have presented, prominently, the symptoms of typhus rather than those of enteric typhoid fever. That is, the first stage has been accompanied by more intense headache; more restlessness; more frequent pulse and respiration; often a semblance of constipation; a thick whitish coat on the tongue, with less tendency to change to red and dry; and the much more frequent appearance of a mulberry or flea-bite rash early in the second stage. The apparent torpor of the bowels, coupled with the coated tongue and intense headache, in the early stage, has often led the patient, and sometimes under the direction of his physician, to use active cathartics and, in a few instances, emetics. I have known of no instance in which such a course has not been productive of very bad consequences. It often induces an early and excessive prostration, coupled with a fixed congestion of the intestinal mucous membrane, greatly favoring softening of the membrane and intestinal *hemorrhage* in the more advanced stage of the fever. Several cases have come under my observation illustrating this tendency.

A few weeks since, I was called to see a young man, in consultation with his attending physician, who had been attacked severely with typhus fever. The bowels not being loose in the early stage, the physician gave him several compound cathartic pills, which did not seem to operate harshly, but soon after a pitcher of lemonade was prepared with half an ounce of chlorate of potassa in it, intending to have the patient use it sparingly as a drink. The nurse, however, allowed the patient free access to the pitcher, and he drank its contents all in one night. This was immediately followed by such copious intestinal evacuations, some of them tinged with blood, that the patient was almost hopelessly prostrated in a few hours and died during the succeeding week.

Another case was that of a man, aged about 25 years, who had recently come to this city. He first called to consult me at my office. I noticed, at once, an unusually haggard expression of countenance; tremulousness of the lips and tongue; a very soft and frequent pulse; with unsteadiness of gait in walking. I learned that he had been laboring under all the symptoms of the first stage of continued fever, for ten days, during which time he had taken repeated doses of active physic, to remove what he called "biliousness." I explained to him the nature of his sickness, prescribed some anodyne and astringent medicine to check the excessive intestinal discharges, and urged him to keep quiet in bed. He persisted, however, in keeping up three or four days longer, at the end of which time I found him in bed, with the same tremulous lips and tongue; the same haggard countenance; a still weaker pulse; and a peculiar tawny hue of the skin, as though the hematine of the red corpuscles had become diffused in the serum of the blood and stained all the tissues. His mind was dull, hearing impaired, urine scanty and turbid, and bowels still moderately loose. In a few days, petechial spots appeared over the chest and abdomen, and, in about ten days from the time he took his bed, he passed at once a chamber-vessel half full of dark, offensive, and partially coagulated blood. The discharge was repeated three or four times within thirty-six hours, producing collapse and death.

The last case I shall mention, was a young man, aged about 23 years, sanguine temperament, and usually in good health. While on a business tour in the country, during the latter part of August and first part of September, he was taken with the usual symptoms of the forming stage of continued fever, viz.: severe pain in the head, back, and limbs; loss of appetite; furred tongue; dryness of the skin and mouth, &c. On arriving at one of the towns on the Mississippi river, he felt so unwell that he was induced to take a warm foot-bath, accompanied by an *emetic*, and followed by an active cathartic. The remedies produced excessive vomiting and purging, but in no degree diminished the headache or fever. With some assistance on the way, he reached his home in this city on the third day after he took the evacuants. His face was flushed; eyes watery and dull; skin dry and moderately hot; tongue covered with a white fur and red at the edges; lips and mouth dry; pulse 110 per minute, small and weak. He complained of a general sense of heaviness and oppression; constant thirst; and severe dull pain, with a sense of dizziness through the whole head, but more severe in the cerebellum and base of the brain. Since he took the emetic and cathartic, three days previously, he had retained nothing on his stomach, more than a very few minutes. A single spoonful of cold water would be promptly ejected by vomiting. I endeavored to overcome the irritability of the stomach by giving a solution of bicarbonate of soda, one drachm, and sulphate of morphia, one grain, in two ounces of water, in doses of a teaspoonful every hour. Sinapisms were also applied to the epigastrium, and cold applications to the head. On the second day, finding the irritability of the stomach only partially allayed, while all the other symptoms were unchanged, I gave, in addition to the soda and morphia, a powder of calomel, 2 gr., every four hours, until it should induce a movement of the bowels. I did this because the continued intense suffering in the head, with fulness of the temporal and carotid arteries, and heat above the rest of the body, caused a fear that a low grade of meningeal inflammation might complicate the case. After four powders had been taken the bowels moved, and the irritability of the stomach was so far relieved that liquids were retained, if taken in small quantities, but the pain and sense of confusion in the head continued unabated, and there was much mental wandering. The pulse was frequent, small, and weak; respiration hurried; some sub-sultus. I ordered a solution of permanganate of potassa, one grain to the ounce of water, to be taken in doses of a tablespoonful every three hours, and a tablespoonful of thin milk porridge every hour. On the following day, finding less heat in the head, but more sub-sultus and mental wandering, I gave one grain of valerianate of quinine in sugar-coated pill, between each of the doses of permanganate, and doubled the quantity of milk porridge for nourishment. He continued this treatment three days, during which the general symptoms underwent but little change, except that the pulse became a little less frequent, the fever less active, and a characteristic typhus eruption appeared quite copiously over the trunk of the body. The hearing also became dull, and a large bullæ or vesicle, filled with purple or bloody serum, appeared on the outside of the right thigh, and another on the scrotum, while the bowels became slightly relaxed. Owing to these symptoms, an emulsion of oil of turpentine and tincture of opium was given in the place of the solution of permanganate of potassa, and the doses of valerianate of quinine were increased to two grains. Under these remedies, with as much simple nourishment as his stomach would bear, he continued to progress favorably, though changing but slowly, for three days longer, when there occurred one pretty copious discharge of dark blood from the bowels. I immediately gave him a powder containing tannate of quinine, 2 grs., pulv. geranium root, 5 grs., and pulv. opium, 1 gr., and repeated it every two hours, with a teaspoonful of the turpentine and laudanum emulsion between. After three doses had been given, and no movement of the bowels occurring, the interval between the doses of medicine was lengthened to two hours. When twenty-four hours had elapsed without further evacuations, the powders were discontinued, and the valerianate of quinine again given in their place.

From this time, the patient began slowly to convalesce, and has since fully recovered. These cases strongly illustrate the evil effects of active purging in the early stage of continued fever. Such practice not merely prostrates the patient, but it directly increases that irritability of the mucous membrane with its aggregate glands of Peyer, which so generally constitutes an important pathological condition in all varieties of continued fever. I think I have never known a case of typhoid or typhus fever, in which active emetics or purgatives were used in the early stage, that did not run a protracted and dangerous course.

